# Anti-Inflammatory Components of *Artemisia scoparia* Alleviate Ulcerative Colitis: An Integrated Study Combining UPLC-Q-Exactive Orbitrap MS/MS, Network Pharmacology, Molecular Docking, and In Vivo/In Vitro Validation

**DOI:** 10.3390/foods15152622

**Published:** 2026-07-27

**Authors:** Panpan Zhang, Xinyu Xu, Yongjie Chen, Yizhen Suo, Yuhong Zhang

**Affiliations:** 1 College of Food and Health, Northeast Forestry University, Harbin 150040, China; zhangpanpan0610@163.com (P.Z.); 18800463554@163.com (X.X.); chenyj2599@163.com (Y.C.); syzo7302014@163.com (Y.S.); 2 Key Laboratory of Forest Plant Ecology, Ministry of Education, Northeast Forestry University, Harbin 150040, China

**Keywords:** *Artemisia scoparia*, ulcerative colitis, UPLC-Q-Exactive Orbitrap MS/MS, network pharmacology, molecular docking

## Abstract

*Artemisia scoparia* is a classic medicinal and edible plant with great exploitation potential in anti-inflammatory research. This study aimed to explore its anti-ulcerative colitis bioactive constituents and corresponding mechanisms of action. A total of 15 flavonoids and coumarins were identified by macroporous resin purification combined with ultra-performance liquid chromatography coupled to UPLC-Q-Exactive Orbitrap MS/MS. Seven key bioactive compounds (eupatilin, cirsimaritin, quercetin, scoparone, 7-hydroxycoumarin, scopoletin and esculetin) and six core targets (TNF, AKT1, SRC, EGFR, HSP90AA1 and ESR1) were further screened via network pharmacology. Subsequent molecular docking analysis verified that flavonoids exhibited markedly stronger binding affinity to target proteins than coumarins. In vitro cellular experiments revealed that eupatilin exhibited the strongest anti-inflammatory activity among all key components. In vivo animal assays further validated that eupatilin alleviated colonic injury and markedly reduced the levels of pro-inflammatory cytokines TNF-α, IL-1β, and IL-6 in mice with DSS-induced ulcerative colitis. This work provides vital experimental evidence for the development and utilization of anti-ulcerative colitis ingredients from *A. scoparia*, and validates its promising health-related application value in the food industry.

## 1. Introduction

Ulcerative colitis is a highly prevalent inflammatory bowel disease worldwide [1], and its exact pathogenesis remains incompletely understood; it is currently attributed to the interplay of multiple factors including environmental, genetic, immune, and gut microbiota influences [2]. Common anti-inflammatory drugs, including non-steroidal anti-inflammatory drugs (NSAIDs), corticosteroids, etc., can cause side effects such as gastrointestinal discomfort and liver and kidney damage [3]. Therefore, researchers are actively exploring novel anti-inflammatory drugs to reduce these adverse effects.

Plant extracts are rich in active components, primarily including flavonoids, coumarins, terpenes, polysaccharides, and polyphenols. In recent years, plant extracts have shown significant biological activity in terms of their anti-inflammatory effects, which has led to interest in their use in the prevention and treatment of ulcerative colitis [4,5,6]. The fruit extract of *Crataegus monogyna* exerts potent protective effects against acetic acid-induced colitis by regulating oxidative stress and inflammatory responses [7]. Astragaloside IV alleviates ulcerative colitis by regulating the balance of Th17/Treg cells [8]. Acidic polysaccharides extracted from *Moringa* leaves ameliorate DSS-induced ulcerative colitis by targeting the metabolic function of intestinal microbiota [9]. Walnut septum-derived aqueous extract suppresses pro-inflammatory cytokines and the JAK1/STAT3 pathway, thereby repairing damaged intestinal mucosal barrier in DSS-induced ulcerative colitis mice [10].

As a medicine and food homologous plant, *Artemisia scoparia* possesses important application value in traditional diets and health care. *A. scoparia* is widely consumed as a culinary condiment and edible raw material for cold dishes in daily diets. In addition to the traditional effects of clearing heat, removing dampness and relieving bile and yellow [11], *A. scoparia* also exhibited anti-inflammatory, antioxidant and antihypertensive effects with complex and diverse pharmacological mechanisms [12]. *A. scoparia* contains mainly coumarins and flavonoids. Some studies have shown that the coumarins and flavonoids play an important role in anti-inflammatory effects [13]. Among them, total flavonoids from *A. scoparia* exert a protective effect on lipopolysaccharide (LPS)-induced acute lung injury in rats, and the molecular mechanism may be related to the inhibition of the activation of NF-κB and MAPK signaling pathways [14].

Previous research studies have preliminarily demonstrated the anti-inflammatory capacity of *A. scoparia*, yet its anti-ulcerative colitis effect, key active ingredients and concrete mechanisms still await systematic clarification. The aim of this study was to reveal the potential anti-inflammatory mechanism and anti-inflammatory components of *A. scoparia* through UPLC-Q-Exactive Orbitrap MS/MS and network pharmacology. The stability of the binding between the components and targets was verified by molecular docking, and the anti-inflammatory effect and mechanism of key components were verified by cellular and animal experiments. This work provides vital experimental evidence for the development and utilization of anti-ulcerative colitis ingredients from *A. scoparia*, and validates its promising health-related application value in the food industry.

## 2. Materials and Methods

### 2.1. Materials

Macropore adsorptive resin D101 (Shanghai yuanye Bio-Technology Co., Ltd., Shanghai, China); RAW264.7 cells (ICell Bioscience Inc., Shanghai, China); DMEM medium (Corning Inc., New York, NY, USA); fetal bovine serum and PBS (Wuhan Pricella Biotechnology Co., Ltd., Wuhan, China); Nitric oxide (NO) assay kit (Beyotime Biotechnology Inc., Shanghai, China); Mouse IL-10 ELISA kit (Boster Biological Technology Co., Ltd., Wuhan, China); Myeloperoxidase (MPO) activity assay kit, mouse tumor necrosis factor alpha (TNF-α), interleukin-1β (IL-1β), and interleukin-6 (IL-6) ELISA kit (Elabscience Biotechnology Co., Ltd., Wuhan, China); HE staining kit (Biosharp, Beijing Langjieko Technology Co., Ltd., Beijing, China); other reagents (Shanghai Macklin Biochemical Co., Ltd., Shanghai, China).

### 2.2. Component Analysis

#### 2.2.1. Plant Extract Preparation

In October 2022, the aerial parts of *A. scoparia* in the flowering stage were harvested in Harbin (45°48′2″ N, 126°33′1″ E, Harbin, China), dried in the shade and crushed to a particle size of 0.85 mm. The plant material was identified by Prof. Baojiang Zheng (Northeast Forestry University, Harbin, China). A voucher specimen (NFU 2022-3283) has been deposited in the plant herbarium of the Northeast Forestry University. Ultrasonic extraction (80% ethanol, 50 °C) for 1.5 h (400 W, 1 g:20 mL) was performed, and filtration residue for secondary extraction was obtained. Ethanol crude extract was concentrated under reduced pressure by combining filtrate. An appropriate amount of ethanol crude extract was dissolved in water to become a suspension, extracted with n-hexane until the solvent became colorless, and ethyl acetate extract (EAC) was obtained from the remaining solution [13,15]. EAC was separated by macropore adsorptive resin D101 with different concentrations (0, 10%, 30%, 50%, 70%, 90%, 100%) of ethanol. The elution volume was 5 times the column volume, and the elution flow rate was 1 drop/s. The eluted solution was subjected to vacuum rotary evaporation to obtain components E1–7.

#### 2.2.2. Purity, Antioxidant and Anti-Inflammatory Activities

The determination of flavonoid and coumarin purity, as well as antioxidant (DPPH^−^ and ASBT^+^ scavenging capacity) and anti-inflammatory (LOX scavenging capacity) activities of each extract fraction, strictly followed the analytical procedures reported by Zhang et al. [13]. Detailed step-by-step protocols for both quantification assays are provided in Supplementary Materials S1.1–1.4.

#### 2.2.3. UPLC-Q-Exactive Orbitrap MS/MS

HPLC analysis was performed on a Thermo Ultimate 3000 HPLC platform (Thermo Fisher Scientific, Waltham, MA, USA). The conditions for the chromatography were as follows: use a Hypersil-Gold Vanquish column (1.9 μm, 100 mm × 2.1 mm); the column temperature is 40 °C; the sample volume is 2 μL; the mobile phases are A 0.1% acetic acid (water acidified to 0.1% with acetic acid) and B acetonitrile; the flow rate is 0.35 mL/min with the following solvent gradient: 0–1 min, 2% B; 1–9 min, 2–98% B; 9–12 min, 98–2% B; 12–15 min, 2% B.

The UPLC system was coupled to a Q-Exactive Orbitrap MS/MS (Thermo Fisher Scientific, Waltham, MA, USA) [16]. Both negative (−) and positive (+) ESI modes were conducted for accurate mass measurements. The conditions for the mass spectrometry were as follows: data were collected in full ms/dd-MS^2^ scanning mode with MS^1^ resolution of 70,000 and MS^2^ resolution of 17,500; the voltage was 3.5 kV (+) and 3.2 kV (−); the evaporation temperature was 350 °C; the capillary temperature was 320 °C; the sheath gas flow rate was 40 arb; and the auxiliary air flow rate was 10 arb. The mass spectrometry data were analyzed using Xcalibur 4.1 (Thermo Fisher Scientific, Waltham, MA, USA).

### 2.3. Network Pharmacology

#### 2.3.1. Gene Target Collection

Canonical SMILES of bioactive compounds were retrieved from PubChem, then imported into SwissTargetPrediction to predict compound-related targets. Disease-associated inflammatory targets were collected from TTD, GeneCards and OMIM databases. Venny 2.1 (CNB-CSIC, Madrid, Spain) was applied to screen overlapping targets between compound and disease datasets.

#### 2.3.2. Protein–Protein Interaction (PPI) Networks

The targets were imported into the STRING database (http://www.string-db.org/ (accessed on 27 September 2024).) to obtain PPI. The PPI plots were beautified using Cytoscape 3.10.0 software (Institute for Systems Biology Inc., Seattle, WA, USA).

#### 2.3.3. GO and KEGG Enrichment

GO enrichment analysis and KEGG pathway enrichment analysis of the obtained potential action targets were performed using the DAVID database (https://david.ncifcrf.gov/summary.jsp (accessed on 27 September 2024).) with *p* < 0.05 as the filtering condition. GO enrichment histograms (top 10) and KEGG enrichment bubble plots (top 25) were plotted through the microbiology platform (https://www.bioinformatics.com.cn (accessed on 27 September 2024).).

#### 2.3.4. Component-Target-Pathway (C-T-P) Topological Network

A C-T-P network was constructed based on Cytoscape 3.10.0 software (Institute for Systems Biology Inc., USA). The size of the degree value of all nodes in the network was calculated to filter out the top-ranked targets. Targets with large degree values are likely to be core targets.

### 2.4. Molecular Docking

Chemical structures of active ingredients were retrieved via PubChem, while target protein structures were downloaded from UniProt. MGLTools 1.5.7 (Guangzhou, China) was adopted to preprocess all molecules, including eliminating water molecules, adding hydrogen atoms, computing partial charges and merging nonpolar hydrogens. Molecular docking simulation was carried out with AutoDock Vina 1.1.2 (CCSB, La Jolla, CA, USA), and the optimal conformer with the highest binding affinity was screened for graphical visualization via PyMOL 2.6.0 software (DeLano Scientific LLC, Palo Alto, CA, USA).

### 2.5. RAW264.7 Cell Experiments

#### 2.5.1. Cell Proliferation Assay

The effects of components on cellular activity were analyzed using the CCK8 method [17]. For the control group (A1), 100 μL complete medium (89% DMEM basal medium, 10% fetal bovine serum, 1% penicillin-streptomycin) was added, and for the sample group, 100 μL working solution (12.5, 25, 50, 100, 200, 400 μM) was added. Each treatment group (A2) was made up of 3 composite wells. RAW264.7 cells in logarithmic growth phase were taken and inoculated into 96-well plates at 1.5 × 10^4^ cells/well. Treatments were grouped as described above and incubated for 24 h (5% CO_2_, 37 °C). Each well was washed three times with PBS after removing the medium, and medium containing 10% CCK8 was added at to 100 μL/well and incubated for 2 h (5% CO_2_, 37 °C). The absorbance value at 450 nm was detected using an enzyme marker (Infinite M200 PRO spectrophotometer Tecan, Switzerland). The relative cell viability was calculated using Equation (1). A0 is the absorbance of CCK8 reagent and medium only.

Relative cell viability = (A2 − A0)/(A1 − A0) × 100%(1)

#### 2.5.2. Detection of NO, MPO, TNF-α, and IL-10

RAW264.7 cells were pretreated with gradient concentrations of test samples (12.5, 25, 50 μM) and 20 μM DEXA (7.85 μg/mL) for 2 h, followed by 24 h co-incubation with 1 μg/mL LPS. The Griess assay was adopted to quantify intracellular NO levels [18]. Cellular MPO, TNF-α and IL-10 contents were measured by commercial ELISA kits following the manufacturer’s protocols [19].

### 2.6. Animals and Experimental Design

#### 2.6.1. Animal Model and Experimental Grouping

This study involved a total of 24 healthy male C57BL/6J mice (body weight 20 ± 2 g) obtained from the Second Affiliated Hospital of Harbin Medical University. The experimental animals were housed in a specific pathogen-free (SPF, ambient temperature of 22–25 °C and relative humidity of 50–55%) facility at the College of Food and Health Sciences, Northeast Forestry University. After 1 week of acclimation, the mice were randomly divided into four groups (n = 6 each): control group (Control), model group (DSS), positive control group (DSS + 5-ASA, 100 mg/kg), and eupatilin group (DSS + Eup, 20 mg/kg). The experimental protocols were set as follows [20]: The Control group was intragastrically administered an equal volume of normal saline by oral gavage. The DSS group was pretreated with an equal volume of normal saline as the model control group, which was designed to obtain baseline data under identical DSS stimulation and eliminate potential interferences caused by the gavage operation itself. The DSS + 5-ASA group was given freshly prepared 5-ASA solution once daily at a dosage of 100 mg/kg body weight for 7 days, while the DSS + Eup group received freshly prepared eupatilin solution once daily at a dosage of 20 mg/kg [20] body weight for 7 days. Simultaneously, except for the Control group, ulcerative colitis was induced in all experimental groups following previously reported protocols. Briefly, mice were given free access to 3% (*w*/*v*) DSS solution, which was refreshed daily for 7 consecutive days. Mice in the Control group were supplied with deionized water ad libitum. All animals were fasted for 12 h after the last administration and sacrificed via cervical dislocation. Blood samples were collected from the orbital venous plexus, centrifuged at 3000 rpm for 10 min to separate serum, and stored at −20 °C for subsequent detection. The colon and spleen were dissected, residual blood was blotted dry before weighing to calculate organ indexes, and colon length was measured. Segments of colon tissues were harvested and fixed in 4% neutral buffered formalin for histological staining. Considering possible tissue damage or insufficient samples during collection and processing, 6 mice from each group were included in the final statistical analysis.

#### 2.6.2. Calculation of Disease Activity Index (DAI)

From the start of animal experiments, the body weight of mice in each group was weighed and precisely recorded at a fixed time every day. Meanwhile, each mouse was individually examined for diarrhea and bloody feces. Scores corresponding to three indicators (loss of weight, diarrhea, and bloody feces) were assigned strictly following the DAI scoring criteria shown in Table 1, and the Disease Activity Index (DAI) was subsequently calculated in accordance with Equation (2) [21].

DAI = (S1 + S2 + S3)/3(2)

#### 2.6.3. Organ Index Calculation

The body weight of mice was continuously recorded throughout the entire experiment. After euthanasia, the weights of fresh colon and spleen were measured, and the colon index and spleen index were calculated using the following Equations (3) and (4):

Colon index = colon weight/body weight × 100%(3)

Spleen index = spleen weight/body weight × 100%(4)

#### 2.6.4. Measurement of Inflammatory Factors in Mouse Serum

The levels of TNF-α, IL-6, and IL-1β in mouse serum were measured using an ELISA kit (Elabscience Biotechnology Co., Ltd., Wuhan, China) [19].

#### 2.6.5. Histological Examination (H&E Staining)

Colonic tissues fixed in 4% paraformaldehyde were embedded in paraffin and sectioned at a thickness of 4 μm. HE staining of tissue sections was performed strictly in accordance with the manufacturer’s protocols using a commercial staining kit (Biosharp, Beijing Langjieko Technology Co., Ltd., Beijing, China), covering a series of procedures including deparaffinization, staining, water washing, dehydration, clearing and mounting. The stained sections were finally observed and photographed under an optical microscope (JEM1400, Tokyo, Japan).

### 2.7. Statistical Analysis

Data are shown as mean ± SD (n = 3). Significant difference analysis was performed using IBM SPSS Statistics 26 (SPSS Inc., Chicago, IL, USA). One-way ANOVA and Duncan’s test were used to determine differences between groups (*p* < 0.05).

## 3. Results and Discussion

### 3.1. Purity, Antioxidant and Anti-Inflammatory Activities of Extract Fractions

**Figure 1:**
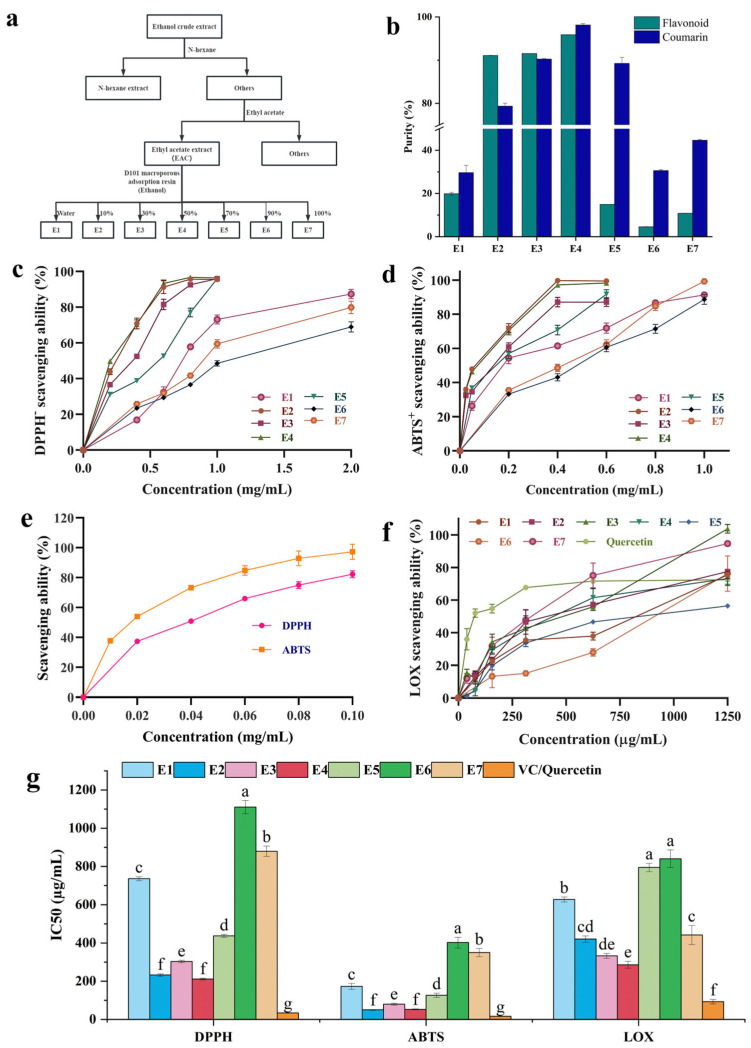
Extraction process (**a**). The purity (**b**), antioxidant activity (DPPH^−^ (**c**) and ABTS^+^ (**d**) scavenging ability, Vc antioxidant activity (**e**)), anti-inflammatory activity (**f**), and IC_50_ values (**g**) of the E1–7 fractions. Data are shown as mean ± SD (n = 3); different letters indicate significant differences, *p* < 0.05.

The separation of EAC by macropore adsorptive resin D101 yielded seven fractions (E1–7) (Figure 1a). E4 had the highest purity of coumarin (98.16%) and flavonoids (95.89%) (Figure 1b, Figures S1 and S2). Studies have confirmed that anti-inflammatory activity is closely related to antioxidant activity [13]. In this study, an activity-guided screening was performed on all fractions using antioxidant and anti-inflammatory activities as evaluation indicators (Figure 1c–g). DPPH^−^ and ABTS^+^ scavenging abilities are widely adopted classic indicators for evaluating in vitro antioxidant activity. LOX inhibitory activity serves as a common biomarker to assess anti-inflammatory effects at the cellular in vitro level.

The difference in maximum tested concentrations among fractions E1–E7 stems from their distinct antioxidant potency. Four fractions (E2, E3, E4, E5) exhibited strong free radical scavenging capacity at low concentrations. Their DPPH^−^ and ABTS^+^ scavenging ability already approached or reached nearly 100% at concentrations ≤ 1.0 mg/mL (DPPH^−^ scavenging ability) and ≤0.6 mg/mL (ABTS^+^ scavenging ability). Further increasing the concentration would not produce measurable increases in scavenging ability, so we terminated the concentration gradient early to avoid redundant saturated data points. E1, E6 and E7 showed relatively low antioxidant activity. Their radical scavenging rates did not reach saturation even at the maximum tested concentrations (2.0 mg/mL for DPPH^−^ scavenging ability, 1.0 mg/mL for ABTS^+^ scavenging ability). The results showed that fraction E4 exhibited the optimal antioxidant and anti-inflammatory activities. The IC_50_ values of fraction E4 in antioxidant and anti-inflammatory activity assays were 302.63 µg/mL (DPPH^−^ scavenging ability), 52.72 µg/mL (ABTS^+^ scavenging ability) and 285.93 µg/mL (LOX scavenging ability), respectively. Therefore, fraction E4 was selected as the target anti-inflammatory fraction for subsequent component analysis.

### 3.2. Component Analysis by UPLC-Q-Exactive Orbitrap MS/MS

Using data from the PubChem website and the literature [22], four coumarins and 11 flavonoids were identified from E4 by UPLC-Q-Exactive Orbitrap MS/MS (Table 2). These components have a variety of pharmacological effects.

All these components exhibit anti-inflammatory activity. Esculetin is a coumarin derivative found in many kinds of natural plant products, with decongestant, anti-inflammatory and anticancer biological and pharmacological properties [23,24]. Scoparone has potential therapeutic benefits for autoimmune and other inflammatory diseases [25,26]. Scopoletin has antibacterial, anticancer, anti-inflammatory and lipid-reducing properties [27,28]. 7-Hydroxycoumarin has antidiarrheal, antioxidant, antibacterial and anti-ulcer properties [29]. Rutin has antioxidant, anti-inflammatory and analgesic properties [30]. Quercitrin has antioxidant, anti-inflammatory, antimicrobial and analgesic properties [31]. Astragalin has anticancer, anti-inflammatory, lipid-reducing, hypoglycemic, neuroprotective and respiratory protective effects [32,33]. Quercetin, as the most common flavonoid, with important antioxidant, anti-inflammatory and antimicrobial properties [34,35]. Isoquercitrin has a higher bioavailability than quercetin, and shows many protective effects both in vivo and in vitro (against oxidative stress, cancer, cardiovascular disease, diabetes and allergic reactions) [36]. Hyperoside has anticancer, anti-inflammatory, antibacterial, antiviral, antidepressant and organ-protective properties [37,38]. Cirsimaritin has anticancer, antibacterial, antidiabetic, antiparasitic, antioxidant and anti-inflammatory properties [39]. Eupatilin has anticancer, antioxidant and anti-inflammatory properties [40,41].

### 3.3. Network Pharmacology Analysis

**Figure 2:**
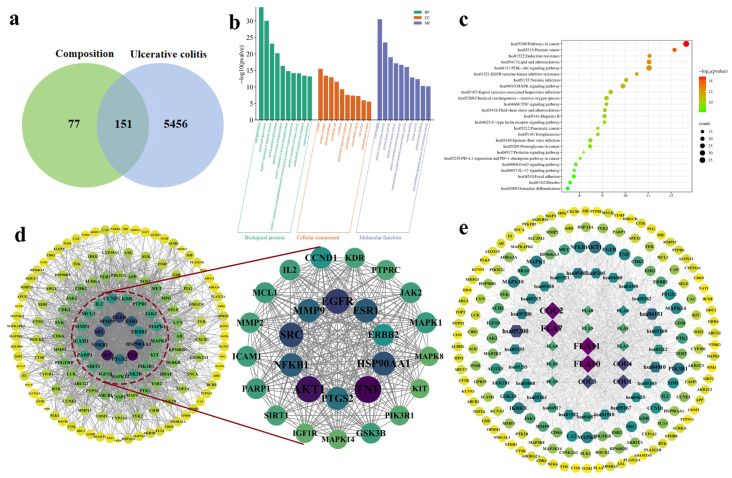
Analysis of ulcerative colitis effects of *A. scoparia* components based on network pharmacology. Component targets and inflammatory targets (**a**); GO enrichment analysis (**b**); KEGG enrichment analysis (**c**); protein–protein interaction (PPI) networks (**d**); component-target-pathway network (C-T-P) (**e**).

Network pharmacology analyzes the multi-component and multi-target mechanism of action of traditional Chinese medicines by integrating multidisciplinary data [42,43]. As shown in the Venn diagram (Figure 2a), 77 unique targets correspond to active ingredients of *A. scoparia*, 5456 disease-related targets are associated with ulcerative colitis, and their intersection yields 151 overlapping candidate targets, which are regarded as the potential therapeutic targets through which *A. scoparia* acts against ulcerative colitis.

To systematically dissect the molecular mechanisms underlying the anti-ulcerative colitis activity of *A. scoparia* active compounds, GO and KEGG enrichment analyses were performed for 151 potential targets. The top 10 biological process (BP), cellular component (CC), and molecular function (MF) entries were used to plot the GO enrichment analysis (Figure 2b). Anti-inflammatory processes are mainly closely related to cellular processes and molecular functions, and compounds may regulate phosphorylation, apoptosis, and inflammatory responses through outer plasma membrane receptors to exert preventive and therapeutic anti-inflammatory effects. The KEGG enrichment results (Figure 2c) showed that the anti-inflammatory effects were significantly enriched in signaling pathways: pathways in cancer, prostate cancer, endocrine resistance, PI3K-Akt signaling pathway and lipid and atherosclerosis. The core target genes (TNF, AKT1, EGFR, SRC, HSP90AA1, ESR1) in PPI networks may be the most important potential targets for prevention and treatment of ulcerative colitis (Figure 2d).

In the C-T-P network, “square” indicates the component, “circle” indicates the target, and “hexagon” indicates the signaling pathway (Figure 2e). FLA11 (eupatilin), FLA10 (cirsimaritin), COU2 (scoparone), FLA7 (quercetin), COU4 (7-hydroxycoumarin), COU1 (esculetin), and COU3 (scopoletin) are larger nodes, and can be regarded as the key components of *A. scoparia* for the prevention and treatment of inflammation. Pathways in cancer, the PI3K-Akt signaling pathway, and the MAPK signaling pathway had larger nodes, and can be regarded as important signaling pathways for anti-ulcerative colitis. The PI3K-Akt and MAPK pathways serve as core signaling pathways for the prevention and treatment of ulcerative colitis. Numerous studies have validated that various natural flavonoids and coumarins alleviate ulcerative colitis symptoms by modulating these two pathways [5,44,45].

As a highly conserved cellular signaling cascade, the MAPK pathway is extensively involved in regulating diverse physiological and pathological processes. The MAPK family consists of subfamilies including ERK, p38 MAPK and JNK [46]. By modulating immune cell activation, cytokine secretion and the production of inflammatory mediators, MAPK signaling participates in the regulation of acute and chronic inflammation [47]. Phosphorylated p38 MAPK is highly overexpressed in the colonic mucosa of ulcerative colitis mice, accompanied by massive secretion of TNF-α and IL-1β, which is closely correlated with intestinal injury [48]. Targeted inhibition of the MAPK pathway exerts synergistic anti-colitis effects through multiple mechanisms. It reduces pro-inflammatory cytokines, activates EGFR to repair intestinal epithelium, and remodels gut microbiota by enriching beneficial bacteria such as Bifidobacterium and Lactobacillus [49,50,51].

The PI3K/Akt pathway regulates inflammatory response, oxidative stress, apoptosis, autophagy, intestinal barrier integrity and gut microbial homeostasis. Its abnormal activation induces NF-κB signaling through IκB phosphorylation, disturbs the balance of pro- and anti-inflammatory cytokines and aggravates intestinal inflammatory injury [52]. Additionally, it modulates apoptotic molecules, including Bcl-2 and caspase-3, to alter intestinal barrier stability [53]. Accumulating studies reveal that PI3K/Akt signaling restores the abnormal expression of tight junction proteins and mucins in ulcerative colitis models [54], and indirectly optimizes gut microbiota composition by regulating bile acids and short-chain fatty acids. Hence, targeted regulation of the PI3K/Akt pathway represents an effective strategy for alleviating ulcerative colitis [55].

The results of C-T-P network analysis indicate that the prevention and treatment of inflammation by *A. scoparia* are characterized by multi-component, multi-target and multi-pathway effects. C-T-P network analysis revealed that these core phytochemicals share multiple overlapping targets within inflammatory signaling cascades, laying a theoretical foundation for their potential synergistic effects. In addition, abundant published phytopharmacological studies have validated that multiple coexisting compounds exert synergistic anti-inflammatory activities, which can be attributed to their capacity to modulate multiple signaling pathways, diverse cell types and various inflammatory biomarkers simultaneously [56]. Co-administration of resveratrol (120 mg/kg per day) and quercetin (240 mg/kg per day) attenuated high-fat diet-induced circulating inflammatory markers, including TNF-α, IL-6 and monocyte chemoattractant protein-1, in rats [57]. Moreover, the combination of resveratrol and quercetin (both at 4 mg/kg per day) synergistically reversed the altered expression of inflammation- and immune-related genes induced by high-fat diet in mice, proving superior to single compound treatment [58]. Similarly, the combined use of curcumin and resveratrol exerted synergistic anti-inflammatory effects both in vitro and in vivo [59,60].

### 3.4. Molecular Docking Analysis

**Figure 3:**
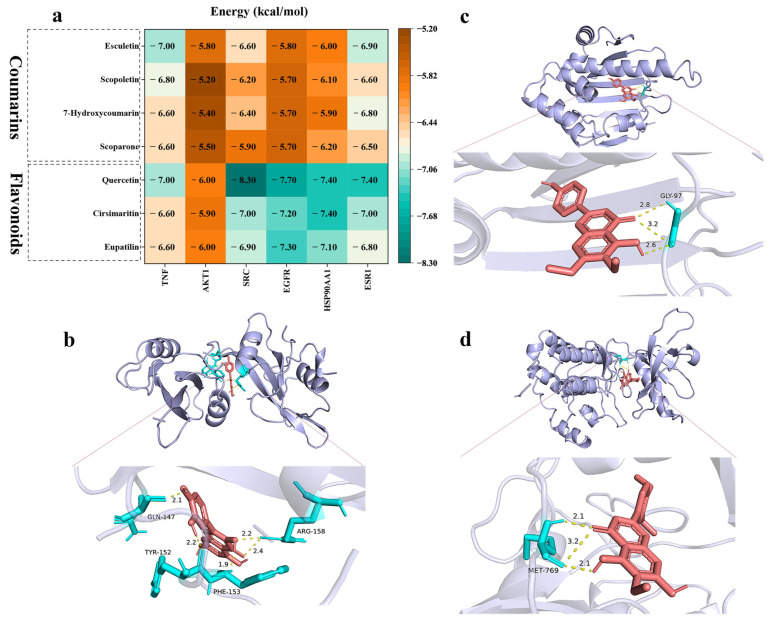
Heat map of molecular docking binding energies (**a**). Molecular docking visualized: quercetin with SRC (**b**); cirsimaritin with HSP900AA1 (**c**); eupatilin with EGFR (**d**).

To investigate the mechanism of interaction between the compound and the target, the binding energy and mode of action were analyzed by molecular docking [61,62]. The results showed a strong binding affinity between the components and the targets. Overall, flavonoids had a stronger binding affinity to targets than coumarins (Figure 3a). Quercetin binds strongly to SRC (−8.30 kcal/mol) and EGFR (−7.70 kcal/mol). Cirsimaritin binds strongly to HSP90AA1 (−7.40 kcal/mol). Eupatilin binds strongly to EGFR (−7.30 kcal/mol). Components and targets can form stable structures with multiple amino acid residues through hydrogen bonding. Quercetin interacts with SRC via GLN-147, PHE-153, and ARG-158 (Figure 3b). Cirsimaritin interacts with HSP900AA1 via GLY-97 (Figure 3c). Eupatilin interacts with EGFR via MET-769 (Figure 3d).

### 3.5. Anti-Inflammatory Effects of Key Components in LPS-Induced RAW264.7 Cells

**Figure 4:**
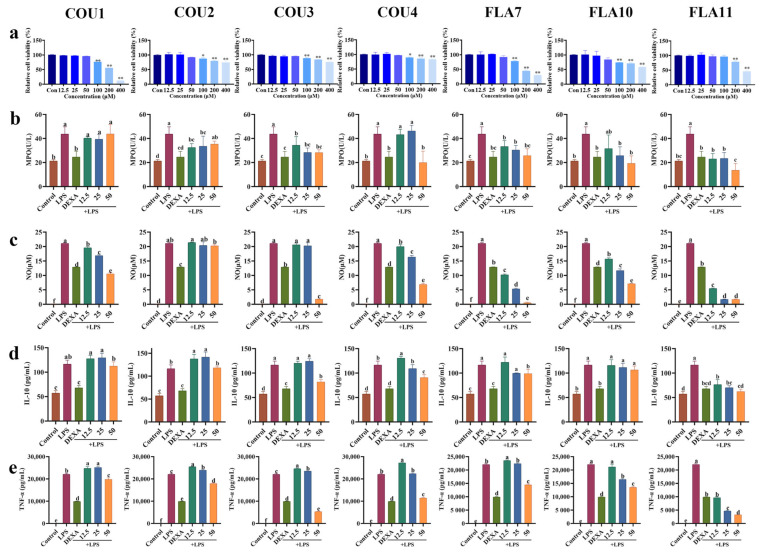
Effects of key components on RAW264.7 cell activity (**a**); data are shown as mean ± SD (n = 3), * *p* < 0.05, ** *p* < 0.01 vs. Con (control). Effects of key components on MPO (**b**), NO (**c**), IL-10 (**d**), TNF-α (**e**) in LPS-induced RAW264.7 cells. Data are shown as mean ± SD (n = 3); different letters indicate significant differences, *p* < 0.05.

To determine the anti-inflammatory activity of the key components (esculetin, scoparone, scopoletin, 7-hydroxycoumarin, quercetin, cirsimaritin, eupatilin), cell activity assays were first performed to select the appropriate working concentrations. The cell activity results (Figure 4a) showed that the cell activity was dose-dependent with respect to the key components. Cell activity was still 95% at concentrations below 50 µM. Therefore, the working concentrations of the key components were set at 50, 25, and 12.5 µM for subsequent experiments. The anti-inflammatory effects of the key components on LPS-induced RAW264.7 cells showed that the key components were able to reduce MPO (Figure 4b), NO (Figure 4c), IL-10 (Figure 4d), and TNF-α (Figure 4e) content in a dose-dependent manner, with the optimal efficacy observed at a concentration of 50 µM. Overall, the results showed that flavonoids had stronger anti-inflammatory ability than coumarins, especially eupatilin (FLA11, 50 µM), which showed the strongest inhibition of MPO, IL-10 and TNF-α.

MPO, NO, IL-10 and TNF-α levels are widely used as biomarkers for cardiovascular disease, diabetes and other inflammation-related diseases [63,64,65]. MPO enhances the inflammatory response by attacking and killing invading microorganisms, primarily through the generation of chlorides and other reactive oxidants [66]. During inflammation, macrophages and other immune cells produce large amounts of NO to fight pathogens, but excess NO can react with superoxide anion to form peroxynitrite, a powerful oxidant that can cause cell and tissue damage and exacerbate inflammation [67]. TNF-α is a major pro-inflammatory cytokine that promotes the inflammatory process and immune response [68]. IL-10 is an anti-inflammatory cytokine that inhibits inflammation and promotes tissue repair. In one study, it was observed that when LPS-stimulated RAW264.7 cells were treated with AGBP-A, IL-10 levels increased and then decreased significantly [69]. The IL-10 level was significantly elevated, which may be due to the abnormal immune response triggered by LPS [70]. However, another study observed that IL-10 levels in LPS-stimulated RAW264.7 cells were first reduced and reversed after emodin treatment [71]. These studies showed that IL-10 levels did not always increase under treatment, and different treatment conditions led to different IL-10 level results.

As a flavonoid compound, eupatilin has been demonstrated to exert anti-inflammatory activity in previous studies. Eupatilin suppresses LPS-induced inducible nitric oxide synthase (iNOS) expression and NO production in a dose-dependent manner, and downregulates the expression of LPS-induced inflammatory mediators and pro-inflammatory cytokines (TNF-α, IL-1β and IL-6), which is consistent with the findings of the present study [72]. Eupatilin markedly inhibits the expression and secretion of pro-inflammatory cytokines (TNF-α, IL-1β and IL-6) both in vitro and in vivo, thereby alleviating allergic inflammatory responses [40]. Oral administration of extracts containing eupatilin and quercetin-3-β-D-glucuronopyranoside ameliorates colonic injury and inflammatory responses in a dose-dependent fashion by suppressing neutrophil activation and oxidative stress [73]. Eupatilin exhibited the most potent anti-inflammatory effect in LPS-induced RAW264.7 cells (Figure 4). On this basis, we further investigated its effects on DSS-induced ulcerative colitis. Eupatilin exerted the most potent anti-inflammatory activity in LPS-induced RAW264.7 cells, and it can be further investigated as a core bioactive constituent of *A. scoparia* against ulcerative colitis.

### 3.6. Eupatilin Alleviates DSS-Induced Ulcerative Colitis

**Figure 5:**
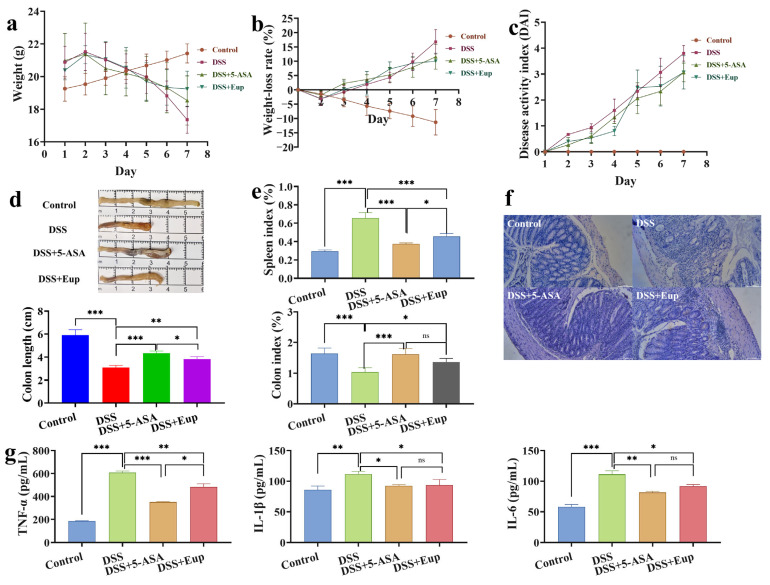
Eupatilin alleviates DSS-induced ulcerative colitis in mice. Body weight changes in mice during modeling (**a**); Body weight loss rate (**b**); DAI score (**c**); Representative macroscopic images of colon tissues and colon length (**d**); Spleen index and colon index statistics (**e**); H&E staining of colon tissue sections (**f**); Levels of pro-inflammatory cytokines TNF-α, IL-1β and IL-6 in colon tissues (**g**). Data are shown as mean ± SD (n = 3), * *p* < 0.05, ** *p* < 0.01, *** *p* < 0.001, ^ns^ *p* > 0.05.

Animal experiments were performed to investigate the ameliorative effect of eupatilin on DSS-induced ulcerative colitis in mice (Figure 5). The results confirmed that the DSS treatment successfully established an acute ulcerative colitis mouse model. Compared with the normal control group, DSS-treated mice exhibited continuous body weight loss, an elevated weight loss rate, and markedly increased DAI scores, accompanied by splenomegaly, shortened and congested colons, severe pathological damage to colonic mucosa, and massive accumulation of pro-inflammatory cytokines, including TNF-α, IL-1β and IL-6, in colon tissues. Intervention with eupatilin significantly reversed all the above pathological manifestations: it effectively alleviated body weight loss (Figure 5a,b), reduced DAI scores (Figure 5c), relieved compensatory splenic enlargement, restored colon length, and ameliorated tissue injuries such as mucosal erosion and inflammatory cell infiltration (Figure 5d–f). Meanwhile, eupatilin remarkably suppressed the secretion of the three key pro-inflammatory cytokines in colon tissues, and its overall protective effect was comparable to that of the positive control drug 5-ASA with no significant intergroup differences (Figure 5g).

The pathological features of the DSS-induced ulcerative colitis model highly recapitulate those of human ulcerative colitis. As a first-line clinical therapeutic agent, 5-ASA served as a positive control to intuitively validate the in vivo anti-colitis activity of eupatilin. This study verified that eupatilin could alleviate systemic inflammatory symptoms and repair the intestinal mucosal barrier, and its protective effect was closely associated with the inhibition of the inflammatory cascade triggered by TNF-α, IL-1β and IL-6, which was consistent with the findings reported by Zhou et al. [20]. Eupatilin exerts intestinal protective efficacy comparable to that of the clinical drug, suggesting its great potential for developing intestinal-repairing functional foods.

Accumulating evidence has demonstrated that numerous natural plant extracts and monomeric phytochemicals exert anti-colitis effects by regulating inflammatory signaling pathways, suppressing pro-inflammatory cytokines and restoring intestinal barrier integrity [4,44,74]. As a pharmacologically vital flavonoid widely distributed in tea, broccoli and other plants, kaempferol can reduce the levels of various inflammatory mediators (IL-6, IL-1β, TNF-α, etc.) in colonic mucosa and upregulate TFF3 to repair intestinal epithelium, thereby alleviating DSS-induced ulcerative colitis [75]. Related studies have also verified that kaempferol and its glycoside derivatives exert intestinal protective effects by remodeling gut microbiota and inhibiting the TLR4/NF-κB signaling pathway [76]. Studies have found that rutin alleviates DSS-induced ulcerative colitis primarily by inhibiting the overactivated PI3K/Akt/GSK3β/MAPKs/NF-κB and p38/MK2 signaling pathways [77]. It can reduce disease activity, restore colon morphology and intestinal epithelial integrity, and downregulate oxidative stress and inflammatory markers as well as regulate the expression of tight junction- and mucus-related genes. As a naturally occurring coumarin abundant in *Phaseolus vulgaris* L., 7-hydroxycoumarin possesses antitumor, anti-inflammatory and antioxidant bioactivities [78]. Relevant animal studies have verified its dose-dependent protective effects against DSS-induced ulcerative colitis via lowering pro-inflammatory cytokines, boosting antioxidant capacity, restraining MAPK pathway activation and restoring gut microbial homeostasis [79,80].

### 3.7. Limitations

Several limitations of this study should be noted. First, the potential molecular mechanisms by which phytochemicals exert anti-inflammatory effects were only predicted via network pharmacology analysis. The screened core targets and signaling pathways have not been verified at the molecular level, and further molecular biological and pharmacological experiments are required for subsequent validation. Second, the synergistic anti-inflammatory effects among various phytochemicals were only preliminarily supported by overall phenotypic data. Rigorous controlled experiments based on cellular and animal models are needed to further clarify the synergistic mode of these multiple components. Third, this study merely quantified the expression levels of representative pro-inflammatory factors in colon tissues and serum, without systematic analyses of the gut microbiome and metabolome. Considering that gut microbiota and their metabolites exert critical regulatory functions in the initiation and progression of inflammatory bowel disease, follow-up research combining gut microbial sequencing and untargeted metabolomics can be conducted to thoroughly elucidate the intrinsic anti-inflammatory mechanism of this plant extract and its active constituents.

## 4. Conclusions

In this study, the anti-inflammatory fraction of *A. scoparia* extract was screened out using an anti-inflammatory activity-guided strategy, and chemical constituents were analyzed by ultra-high performance liquid chromatography coupled with UPLC-Q-Exactive Orbitrap MS/MS. Combined with network pharmacology and molecular docking techniques, the key anti-inflammatory components in *A. scoparia* were screened, and their potential anti-inflammatory mechanisms were explored. The cellular assay showed that the key components had anti-inflammatory effects on LPS-induced RAW264.7 cells by reducing the cellular levels of MPO, NO, IL-10 and TNF-α. In particular, eupatilin (50 µM) had a strong inhibitory effect on MPO, IL-10 and TNF-α, even better than that of the positive control. Moreover, in vivo animal assays validated that eupatilin alleviated DSS-induced ulcerative colitis in mice. Collectively, multiple bioactive components in *A. scoparia* exert anti-ulcerative colitis effects through the synergistic regulation of inflammatory pathways and suppression of pro-inflammatory factor release. As one of its core anti-inflammatory ingredients, eupatilin lays a solid theoretical foundation for intervention in ulcerative colitis.

## Figures and Tables

**Table 1:** Score criteria for DAI.

Score	S1	S2	S3
	Loss of Weight	Diarrhea	Bloody Feces
0	<1%	Normal	Normal
1	1–5%	Loose stool without perianal adhesion	Brown stool
2	5–10%	Loose stool with perianal adhesion	Reddish stool
3	10–15%	Diarrhea	Mildly bloody stool
4	>15%	Severe diarrhea	Bloody stool

**Table 2:** Flavonoids and coumarins in *A. scoparia* identified by UPLC-Q-Exactive Orbitrap MS/MS.

No.	Name	Molecular Formula	+/−	Theoretical Value	Actual Value	Time/min (ppm)	Fragment Ion Peaks (MS^2^)
COU1	Esculetin	C9H6O4	+	179.0338852	179.03293	4.38 (−0.955)	123.04355; 133.02766; 151.03827; 135.04333
COU2	Scoparone	C11H10O4	+	207.0651853	207.06396	5.47 (−1.265)	207.06392; 151.07442; 191.03265; 192.04053; 179.06918
COU3	Scopoletin	C10H8O4	+	193.0495353	193.04843	4.87 (−1.105)	133.02766; 178.02498; 94.04124; 122.03572; 137.05896
COU4	7-Hydroxycoumarin	C9H6O3	+	163.0389706	163.03786	5.09 (−1.111)	135.04327; 107.04876; 119.41496
FLA1	Rutin	C27H30O16	−	609.1461079	609.14514	4.69 (0.129)	300.02667; 301.03433; 271.02411; 255.02913
FLA2	Isorhamnetin 3-O-glucoside	C22H22O12	−	477.1038492	477.10150	5.02 (−1.252)	314.04245; 271.02417; 285.03967; 243.02899; 315.04996
FLA3	3′-O-Methylquercetin 3-galactoside	C22H22O12	−	477.1038492	477.10266	5.21 (−0.972)	271.02402; 299.01855; 300.02750; 315.04987
FLA4	Quercitrin	C21H20O11	−	447.0932846	447.09381	5.10 (0.441)	301.03397; 300.02698; 271.02411; 243.02869
FLA5	Astragalin	C21H20O11	−	447.0932846	447.09143	5.29 (−1.939)	284.03024; 285.03952; 255.02916; 227.03397
FLA6	Quercetin-7-O-β-D-glucopyranoside	C21H20O12	−	463.0881992	463.09003	6.62 (−2.928)	301.03455; 341.17221
FLA7	Quercetin	C15H10O7	+	303.0499291	303.04813	5.06 (−1.799)	29.04834; 257.04202; 165.01730; 285.03790; 247.05894
FLA8	Hyperoside	C21H20O12	−	463.0881992	463.08553	4.83 (−1.772)	271.02399; 300.02655; 301.03426; 299.02045
FLA9	Isoquercitrin	C21H20O12	−	463.0881992	463.08777	5.07 (0.668)	300.02682; 301.03491; 271.02420; 255.02913; 151.00211
FLA10	Cirsimaritin	C17H14O6	+	315.0863146	315.08432	6.55 (−1.995)	108.02025; 254.05583; 119.04870; 136.01471; 226.06116
FLA11	Eupatilin	C18H16O7	−	343.0823261	343.08023	6.65 (−0.999)	298.01111; 270.01620; 313.03455; 328.05789

## Data Availability

The original contributions presented in this study are included in the article/Supplementary Material. Further inquiries can be directed to the corresponding author.

## Supplementary Materials

The following supporting information can be downloaded at: https://www.mdpi.com/article/10.3390/foods15152622/s1, S1.1: Flavonoid purity; S1.2: Coumarin purity; S1.3: Antioxidant activities; S1.4: Anti-inflammatory activities; Figure S1: Standard calibration curve of lutein for total flavonoid purity; Figure S2: Standard calibration curve of coumarin for total coumarin purity.
