# Supplementary material for: Anti-Inflammatory Components of Artemisia scoparia Alleviate Ulcerative Colitis: An Integrated Study Combining UPLC-Q-Exactive Orbitrap MS/MS, Network Pharmacology, Molecular Docking, and In Vivo/In Vitro Validation
Source: Foods. 2026 Jul 27;15(15):2622. doi: 10.3390/foods15152622 (PMC13465494; doi:10.3390/foods15152622)
Supplement: Supplementary file 1 [file foods-15-02622-s001.zip › foods-4419810-supplementary.pdf]

# Supplementary Materials

## 1 Materials and Methods

### *S1.1 Flavonoid purity*

The flavonoid content was determined by the $NaNO_2$-$Al(NO_3)_3$-NaOH method using lutein as the standard substance with slight modifications. Place 1 mL of the solution to be tested (1 mg/mL) into a 10 mL colorimetric tube, add 0.3 mL of 5% $NaNO_2$ solution, shake well, leave for 6 min, add 0.3 mL of 10% $Al(NO_3)_3$ solution, shake well, leave for another 6 min, add 4 mL of 1 mol/L NaOH solution and dilute to the mark with anhydrous ethanol. Allow to stand for 15 min and read the absorbance at a wavelength of 510 nm without adding the sample as a blank control. The concentrations of the standard curve for lutein were set at 0.1, 0.2, 0.3, 0.4, and 0.5 mg/mL, and both the standard curve and the sample content were calculated from the respective absorbance values.

### *S1.2 Coumarin purity*

Using coumarin as a reference standard, the content of coumarin was determined by the light absorption method. Specifically, 1 mL of the liquid to be tested at a concentration of 0.1 mg/mL was placed into a 10 mL colorimetric tube, and the absorbance at 310 nm was measured in 80% ethanol at a constant volume of 10 mL. The standard curve for coumarin ranged from concentrations of 0.05, 0.10, 0.15, 0.20, and 0.25 mg/mL, and both the standard curve and content were calculated from the respective light absorbance values.

### *S1.3 Antioxidant activities*

$DPPH^{-}$ scavenging capacity was determined using the 96-well plate method. DPPH (2.0 mg) was dissolved in 100 mL anhydrous ethanol by ultrasonication until complete dissolution to obtain the DPPH working solution. Subsequently, 50 μL extracts of different concentrations were mixed with 150 μL of the DPPH working solution (A1), while a control mixture containing 50 μL anhydrous ethanol and 150 μL of the DPPH working solution was prepared (A0). The reaction was carried out in the absence of light for 30 min, after which the absorbance was measured at a wavelength of 517 nm (infinite M200 PRO spectrophotometer Tecan, Switzerland), using vitamin C (VC) as a positive control. Each dataset was obtained from three replicate experiments. The DPPH free radical scavenging rate was determined using formula 1.

$ABTS^{+}$ scavenging capacity was determined using the 96-well plate method. A total of 0.03841 g of ABTS was weighed and dissolved in 10 mL of deionized water, followed by ultrasonication until complete dissolution, resulting in a 7 mmol/L ABTS solution. Additionally, 0.0662 g of $K_2S_2O_8$ was weighed and dissolved in 100 mL of deionized water, followed by ultrasonication until complete dissolution to give a 2.45 mmol/L $K_2S_2O_8$ solution. The ABTS solution and $K_2S_2O_8$ solution were mixed in equal volumes and allowed to react in the dark for 12–16 h to produce a mother liquor, which was then diluted with anhydrous ethanol into a working solution ($OD_{734nm}$ = 0.7 ± 0.02). Extracts (50 μL) of different concentrations mixed with 150 μL ABTS working solution (A1), as well as 50 μL anhydrous ethanol mixed with 150 μL ABTS working solution (A0), were tested for light absorption at

734 nm wavelength for 6 min, with VC as a positive control. The measurements in each group were repeated three times. The ABTS free radical scavenging rate was determined using formula 1.

$$Scavenging\ ability = (1 - A1 / A0) \times 100\% \quad (1)$$

A0: Absorbance of control; A1: Absorbance of the test sample.

***S1.4 Anti-inflammatory activities***

The LOX inhibitory activity of the extracts was evaluated using the FOX method in 96-well plates (Ondua et al., 2019). A 50 μL solution of LOX (200 U/mL, dissolved in 50 mmol/mL pH 7.4 Tris HCl buffer) was mixed with a 20 μL sample solution (plant extract or standard) at 25 ℃ for 5 min, followed by the addition of 50 μL linoleic acid (140 μmol/L, dissolved in 50 mmol/L pH 7.4 Tris HCl) and incubation at 25 ℃ for a further 20 min. The reaction was then stopped, and color development initiated by the addition of 100 μL FOX (30 mmol/L sulfuric acid, 100 μmol/L xylenol orange, 100 μmol/L ferrous sulfate, 90% methanol, and 10% water) at 25 ℃ for 30 min. Absorbance was measured at 560 nm using an enzyme marker. Quercetin was used as the positive control. The measurements in each group were repeated three times. The inhibition rate of lipase was determined using formula 2.

$$Inhibition\ rate\ of\ LOX = (1 - (A1 - A2) / (A3 - A4)) \times 100\% \quad (2)$$

A1: Sample + LOX +linoleic acid + FOX; A2: Sample + Tris HCl +linoleic acid + FOX; A3: Tris HCl + LOX +linoleic acid + FOX; A4: Tris HCl + Tris HCl +linoleic acid + FOX.

## Figures

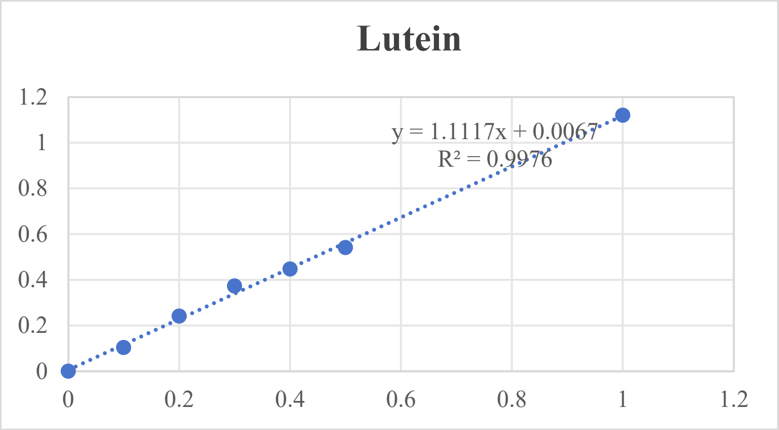


**Figure S1.** Standard calibration curve of lutein for total flavonoid purity.

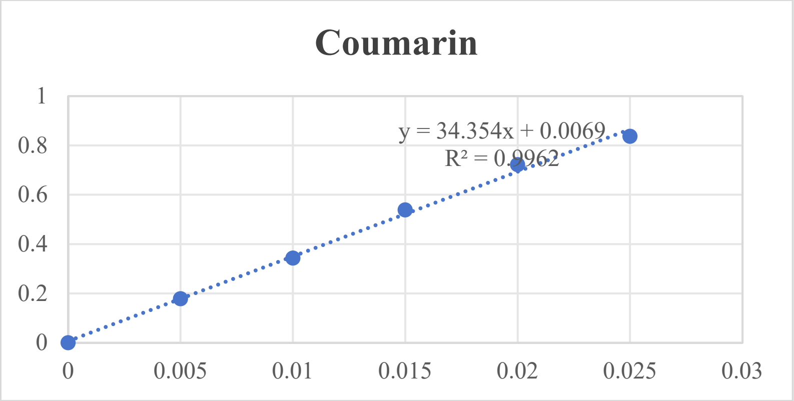


**Figure S2.** Standard calibration curve of coumarin for total coumarin purity.
